# Editorial: New insights into stellate ganglion blockade in basic and clinical studies

**DOI:** 10.3389/fnins.2026.1838108

**Published:** 2026-04-20

**Authors:** Zhi-Ling Guo, Christopher Reist, Shaista Malik

**Affiliations:** 1 Susan Samueli Integrative Health Institute, University of California, Irvine, CA, United States; 2 Department of Medicine, School of Medicine, University of California, Irvine, CA, United States; 3 Department of Psychiatry, School of Medicine, University of California, Irvine, CA, United States

**Keywords:** animal models, autonomic nervous system, physiological responses, stellate ganglion, ultrasound, psychiatric disorders

## Introduction

The autonomic nervous system plays a central role in regulating physiological responses to stress, coordinating cardiovascular function, sleep regulation, immune signaling, and emotional processing. Increasing evidence suggests that dysregulation of autonomic balance, particularly persistent sympathetic activation, contributes to the pathophysiology of multiple psychiatric and medical disorders.

Recognition of the role of autonomic dysfunction has stimulated interest in therapeutic strategies that directly target sympathetic signaling pathways. One such intervention is stellate ganglion blockade (SGB), a procedure that involves injecting local anesthetic around the cervicothoracic sympathetic ganglion. Originally developed to treat sympathetically mediated pain conditions, SGB has increasingly been investigated as a neuromodulatory intervention capable of influencing central neural circuits involved in stress responses and other neural and psychological disorders. The adoption of ultrasound-guided SGB has marked a pivotal advancement, proving to be more effective and safer compared to traditional blind injection techniques (Aleanakian et al., 2020; Li et al., 2022). Despite these advancements, thorough evaluation and broader understanding of SGB's applications remain crucial.

The articles included in this Research Topic reflect the expanding interest in SGB across clinical, translational, and mechanistic domains. Together, they illustrate how sympathetic modulation may influence diverse physiological responses and suggest new directions for research into autonomic regulation.

## Clinical studies of sympathetic modulation

Conditions such as post-traumatic stress disorder (PTSD), generalized anxiety disorder, and sleep disturbances frequently exhibit features consistent with sustained sympathetic hyperactivation. Elevated norepinephrine signaling, exaggerated startle responses, and impaired parasympathetic regulation are commonly observed in these conditions. Early clinical observations describing the use of SGB in PTSD documented rapid reductions in hyperarousal symptoms, irritability, and sleep disturbance. These findings generated interest in the possibility that SGB may influence central neural networks involved in stress responses rather than acting solely as a regional anesthetic technique (Lipov et al., 2012; Mulvaney et al., 2015).

Subsequent investigations have begun to explore this hypothesis in more systematic ways. In a randomized controlled study of patients with generalized anxiety disorder accompanied by sleep disturbance, Liu N. et al. reported that SGB treatment was associated with significant improvements in anxiety symptoms and sleep quality compared with conventional therapy. They also observed measurable changes in circulating neurotransmitters, including reductions in norepinephrine levels and increases in serotonin and neuropeptide-Y, suggesting that SGB may influence neurobiological pathways involved in stress regulation.

In addition to treating established symptoms, Gao et al. have begun exploring whether sympathetic modulation could prevent the development of trauma-related disorders. A randomized clinical trial protocol worked by them examining the use of perioperative SGB in patients undergoing emergency ocular trauma surgery will test the hypothesis that early sympathetic blockade may reduce the risk of developing PTSD following acute traumatic injury. This preventive approach reflects a broader shift in thinking about trauma-related disorders, not simply as psychological responses but as conditions influenced by neurobiological processes involved in fear memory consolidation and autonomic regulation.

## Insights from translational models

While clinical studies provide important evidence of therapeutic effects, translational animal models are essential for understanding the mechanisms through which SGB influences physiological responses.

Recent experimental work has demonstrated that ultrasound-guided SGB can be reliably and safely performed in rodent animal models, producing consistent physiological markers of sympathetic interruption. In this regard, Tran et al. reported that SGB, in rats, slows heart rate and increases heart rate variability. In mice, Liu X. et al. demonstrated that ultrasound-guided SGB leads to decreased heart rate, increased carotid artery blood flow, and thermoregulatory changes in the ipsilateral limb. These findings confirm effective blockade of cervicothoracic sympathetic pathways following SGB under ultrasound guidance in small animal models and provide a platform for investigating the neural, molecular, and genetic consequences of SGB-induced sympathetic modulation. In this respect, for example, (Wang et al. 2025) suggest that modulation of cervicothoracic sympathetic signaling may influence activity within the locus coeruleus-amygdala pathway, a neural circuit critically involved in fear memory consolidation and hyperarousal responses in mice. As such, these and other hypotheses can now be tested further in experimental models.

## Expanding clinical applications

While much of the recent interest in SGB has focused on neurological and psychiatric disorders, emerging reports suggest that sympathetic modulation may influence other physiological reflex responses as well.

Lu et al. describe a patient with severe refractory hiccups successfully treated with SGB combined with phrenic nerve radiofrequency modulation, suggesting a potential role for sympathetic intervention in persistent hiccup reflex circuits. Although based on a single case, this observation highlights interactions between autonomic and somatic neural pathways and suggests that SGB may have therapeutic implications beyond disorders with more established autonomic targets.

Interest in autonomic neuromodulation has expanded further with the recognition that post-viral syndromes may involve persistent dysregulation of autonomic signaling. Many individuals experiencing Long COVID report symptoms such as fatigue, orthostatic intolerance, cognitive impairment, and gastrointestinal dysfunction. These features resemble conditions associated with dysautonomia. Recent clinical observations by Liu and Duricka suggest that autonomic nerve blocks, including SGB, may improve certain Long COVID symptoms. Furthermore, their reports describe the use of autonomic plexus blockade to improve persistent gastrointestinal symptoms, highlighting the role of autonomic signaling in regulating the gut-brain axis. These findings raise the possibility that sympathetic modulation may have broader applications in conditions characterized by post-viral autonomic dysfunction.

## Conclusion and perspectives

The studies presented in this Research Topic highlight the growing recognition of autonomic dysregulation as a common feature across multiple disorders. At the same time, they illustrate how SGB may function as a neuromodulatory intervention that can influence central stress circuits and systemic physiological regulation.

Despite promising findings, additional randomized clinical trials are needed to determine SGB's efficacy across diverse patient populations and to identify optimal treatment protocols. Such large-scale studies are occurring for PTSD, for which SGB is being widely adopted (Hollifield et al., 2026). Utilizing available neuroimaging paradigms and neurophysiological monitoring in these studies will help to elucidate how SGB influences central brain networks involved in stress regulation. Hopefully, this information will be complemented by the continued development of translational models critical for identifying molecular and neural mechanisms linking peripheral autonomic intervention to central neuromodulatory effects. Understanding these mechanisms may ultimately lead to new therapeutic strategies targeting autonomic dysregulation across a wide range of psychiatric and medical disorders. Furthermore , it is of great interest to investigate whether the duration of SGB's efficacy can be extended with a long-acting neuroblocker, such as botulinum toxin.

Additionally, SGB is an area of growing interest in the management of cardiac disorders. Recent case reports suggest that left SGB may alleviate coronary artery spasm, supporting a role for the stellate ganglia in sympathetic innervation of the coronary arteries (Ahmed et al., 2025; Lanza and Shimokawa, 2023). The superior cervical ganglion has also been shown to regulate cardiac sympathetic activity (Shi et al., 2019). However, it remains unclear whether postganglionic sympathetic fibers from the stellate ganglia and the superior cervical ganglion differentially innervate the coronary arteries. Further study is needed to clarify the mechanisms by which these ganglia influence cardiovascular function.
